# Kinetically Controlled Fabrication of Single‐Crystalline TiO_2_ Nanobrush Architectures with High Energy {001} Facets

**DOI:** 10.1002/advs.201700045

**Published:** 2017-04-05

**Authors:** Lisha Fan, Xiang Gao, Dongkyu Lee, Er‐Jia Guo, Shinbuhm Lee, Paul C. Snijders, Thomas Z. Ward, Gyula Eres, Matthew F. Chisholm, Ho Nyung Lee

**Affiliations:** ^1^ Oak Ridge National Laboratory Oak Ridge TN 37831 USA

**Keywords:** defect‐mediated aggregation, high energy {001} facets, kinetic growth control, pulsed laser deposition, TiO_2_ nanostructures

## Abstract

This study demonstrates that precise control of nonequilibrium growth conditions during pulsed laser deposition (PLD) can be exploited to produce single‐crystalline anatase TiO_2_ nanobrush architectures with large surface areas terminated with high energy {001} facets. The data indicate that the key to nanobrush formation is controlling the atomic surface transport processes to balance defect aggregation and surface‐smoothing processes. High‐resolution scanning transmission electron microscopy data reveal that defect‐mediated aggregation is the key to TiO_2_ nanobrush formation. The large concentration of defects present at the intersection of domain boundaries promotes aggregation of PLD growth species, resulting in the growth of the single‐crystalline nanobrush architecture. This study proposes a model for the relationship between defect creation and growth mode in nonequilibrium environments, which enables application of this growth method to novel nanostructure design in a broad range of materials.

## 1 Introduction

1D nanostructures, including tubes, rods, and wires, provide fascinating structure‐dependent properties and a high potential for nanotechnologies and related applications in electronics, information storage, optoelectronics, electrochemistry, and electromechanical devices.^1^, ^2^, ^3^ Due to the structural perfection offered in single crystals, the ability to fabricate single crystalline 1D nanostructures is highly advantageous for developing high performance nanomaterials and devices. Pulsed laser deposition (PLD) is a widely known nonequilibrium growth method for growing epitaxial oxide thin films and superlattices.^4^, ^5^ The growth of atomically flat epitaxial thin films by PLD is kinetically governed by a multitude of atomistic processes, including surface diffusion, nucleation, atom attachment/detachment, and interlayer mass transport.^6^ On the other hand, many growth instabilities, including uncontrolled aggregation, clustering, and step bunching, compete with surface smoothing processes necessary for ideal 2D growth, potentially leading to a rough growth front or inclusion of growth defects.^7^, ^8^ Motivated by the quest for atomically precise heteroepitaxy, extensive efforts have been made on suppressing such growth instabilities. These efforts have revealed the important role of the adatom mobility for 2D epitaxial growth, as determined by the substrate temperature and the reactive gas pressure during growth by PLD.^6^ The incident kinetic energy of the growth species in the laser plume can also be tuned by various means, including laser fluence, substrate‐to‐target distance, and spot size.^9^, ^10^

Many more opportunities could be further envisioned when these growth instabilities are intentionally created and manipulated in order to create unconventional architectures. The ideal 2D growth mode can be readily interrupted by growth instabilities, which cause growth front roughness. When the growth front roughness is extremely enhanced due to limited surface diffusion, a columnar structure will be developed. Kinetic control between growth instabilities and surface equilibration is therefore critical to break down the 2D epitaxy, increasing the possibility of constructing 1D nanostructures. Recent reports of growing highly crystalline 1D nanostructures that exhibit good ionic conductivity in yttria‐stabilized zirconia (YSZ), enhanced low‐field magnetoresistance in La_0.3_Ca_0.7_MnO_3_, and high optical quality in ZnO are good examples of the importance of mastering the growth control and understanding the growth mechanism for synthesizing functional architectures.^11^, ^12^, ^13^, ^14^, ^15^, ^16^, ^17^ For example, Zhou et al. pointed out the role of defects to control morphology of 1D nanostructured BaTiO_3_ films.^12^ However, a comprehensive study of the connection between defects and the growth mode transition from a 2D thin film to a 1D architecture is still missing.

In this report, we focus on 1D nanostructured TiO_2_ because 1D TiO_2_ tubes,^18^, ^19^ wires,^20^, ^21^ rods,^22^, ^23^ and tree nanostructures^24^, ^25^, ^26^, ^27^ have shown superior performance over their 0D and 2D counterparts due to their large specific surface area and excellent electronic charge transport properties useful for many energy and environmental devices, such as photocatalysis, water splitting, photovoltaics, gas sensing, and renewable energy generation and storage.^28^, ^29^ Of the three major polymorphs of TiO_2_, anatase is the most widely explored phase for practical applications.^30^ The equilibrium Wulff crystal shape of anatase TiO_2_, obtained by an analysis of surface energies, is dominated (>94%) by low energy {101} facets.^31^ Both theoretical and experimental studies suggested that the (001) surface is chemically more active than the (101) surface, exhibiting superior performance in many applications, such as photocatalysis, photovoltaics, electrochemistry, water treatment, and energy storage.^32^, ^33^, ^34^, ^35^, ^36^, ^37^, ^38^, ^39^, ^40^ Therefore, synthesis of TiO_2_ nanostructures with a large percentage of reactive {001} facets is highly desired. Solvothermal synthesis using hydrofluoric acid (HF) as a morphology controlling agent is currently the most effective and popular method for stabilizing the {001} facets of anatase TiO_2_.^37^ However, there has been no report on direct synthesis of 1D anatase TiO_2_ nanostructures with a high concentration of the {001} facets using physical vapor deposition method.

In this study, we report single‐crystalline anatase TiO_2_ 1D “nanobrush” architectures fabricated on (001) SrTiO_3_ (STO) substrates within a small window of kinetic growth parameters. The 1D nanobrushes are epitaxially stabilized on a continuous 2D anatase underlayer and feature a large fraction of exposed {001} facets. Based on a systematic study of the microstructural evolution of a TiO_2_ nanobrush film, we describe the growth morphology in terms of defect‐mediated aggregation, stressing the important role of the misfit defects in the heteroepitaxial growth of 1D nanobrush architectures that are generated by operating PLD in the defect producing modality.

## 2 Results

**Figure 1:**
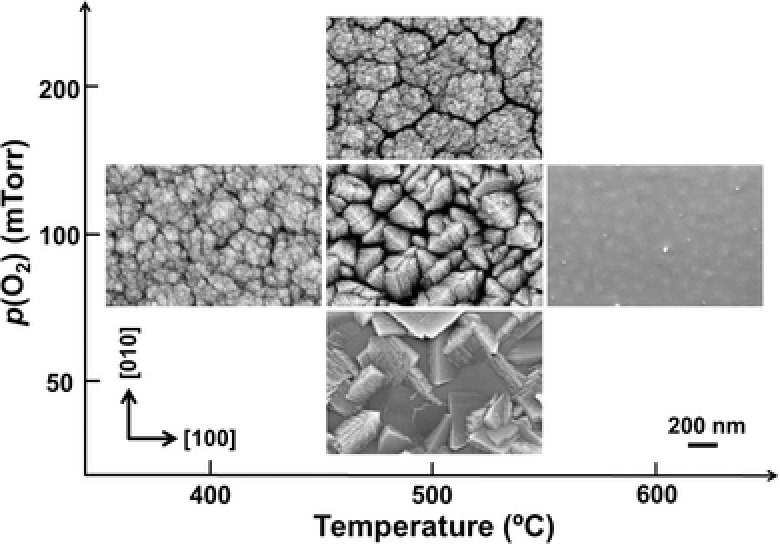
plan view SEM images of TiO_2_ films grown under a wide range of substrate temperature and oxygen pressure, *p*(O_2_), conditions.

The plan view scanning electron microscopy (SEM) images of films in **Figure** **1** illustrate the growth temperature and oxygen background pressure parameter space covered in this work. The growth temperature governs the surface diffusivity of the arriving growth species before they are incorporated into the lattice, while the background pressure modulates the kinetic energy of the growth species and the formation of clusters in the plume. The most porous nanostructure is observed in a sample grown at 500 °C in 100 mTorr O_2_, revealing a spatially separated roof‐like surface morphology (≈200 nm in lateral size) consisting of triangular lamellae that are stacked along the <110> directions. Film densification occurs at a higher substrate temperature of 600 °C or a lower *p*(O_2_) of 50 mTorr. The breakdown of the compact 2D epitaxy that can be seen from the sample grown at 500 °C in 100 mTorr is attributed to the reduced adatom mobility and resultant aggregation that prevails when surface diffusion is suppressed. However, further reduction of the temperature or an increase in oxygen pressure resulted in an undesired cauliflower‐like surface morphology.

**Figure 2:**
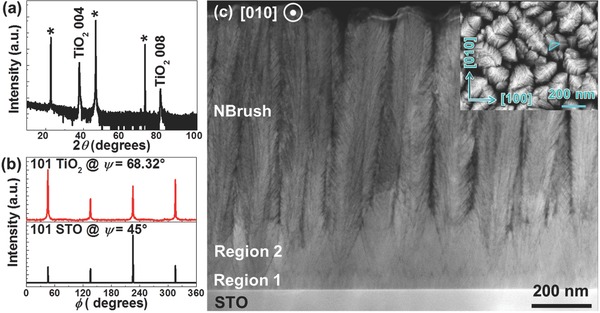
High‐resolution XRD scans of a TiO_2_ nanobrush film: a) XRD θ–2θ scan (* denotes 00*l* peaks from the STO substrate); b) in‐plane XRD φ scans of the TiO_2_ 101 and STO 101 reflections at ψ = 68.32° and 45°, respectively. c) A cross‐sectional STEM image and a plan view SEM image (inset in (c)) of a TiO_2_ nanobrush film.

The morphology of the porous film with a repeated roof‐like surface structure suggested that the film was obviously not continuous. Porosity often originates from the breakdown of 2D epitaxy and results in the formation of polycrystalline or even amorphous phases. Surprisingly, neither impurity phases nor different orientations were detected in X‐ray diffraction (XRD) scans (see **Figure** **2**a), confirming the synthesis of a single‐crystalline nanostructured TiO_2_ film despite its high porosity observed in the SEM image shown in Figure 1. Note that the overall film is porous, but each bristle. The crystallinity of the sample was further evaluated by XRD rocking curve measurements, and the typical full‐width at half‐maximum value of the 004 reflection was ≈0.8°, revealing relatively good crystallinity when considering the overall thickness and porosity. The in‐plane XRD φ scans for the TiO_2_ 101 and STO 101 reflections at ψ = 68.32° and 45°, respectively, confirm the epitaxy of a nanostructured TiO_2_ film on STO with a clear fourfold symmetry as shown in Figure 2b.

The cross‐sectional scanning transmission electron microscopy (STEM) image of this film in Figure 2c illustrates the striking nanobrush structure of vertically aligned 1D microscopic nanobristles. The nanobrush film does not exhibit a uniform structure along the surface normal direction, but is rather composed of three distinct regions. Region 1 is formed by a dense, continuous layer with a thickness of around 75 nm. Region 2 of around 280 nm contains conical “roots” (see below) of the bristles that form the micrometers thick nanobrush region. The boundary between Region 1 and Region 2 is characterized by a sharp zig‐zag structure of darker lines. Each bristle in the nanobrush region contains a large amount of nearly parallel “branches” extending from the core. The bristle diameter averages around 150 nm, and neighboring bristles are separated by voids. Correlating the plan view SEM morphology in Figure 1 with the cross‐sectional STEM observations in Figure 2c reveals that the triangle‐shaped lamellae are in fact the branches extending from the bristle core. Similar cross‐sectional morphology of TiO_2_ hierarchical nanobrush structures have been reported by several groups independently.^24^, ^25^, ^26^, ^27^ However, in these reports, the hierarchical nanostructures are vertically aligned bundles of polycrystalline TiO_2_ nanoparticles as shown by both TEM imaging and XRD scans.^26^, ^27^ Our TiO_2_ nanobrushes are single crystalline throughout the film and are epitaxially connected to the underlying substrate. The ability to synthesize epitaxial nanomaterials provides advantages over conventional polycrystalline or amorphous TiO_2_ nanostructures since properties and performance of materials are strongly dependent upon their crystallinity and orientation.^41^, ^42^

**Figure 3:**
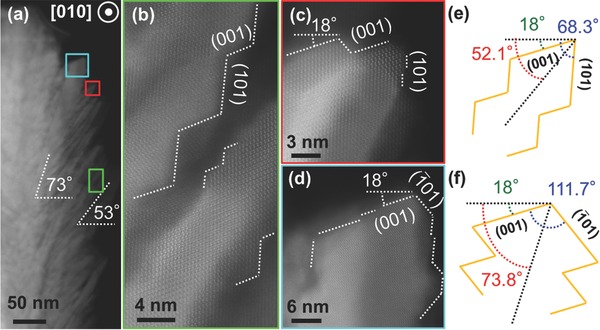
Microstructures of a single TiO_2_ nanobristle and its branches. a) STEM image of a single nanobristle; b–d) HAADF images of the regions in (b) the green rectangle, (c) the red square, and (d) the cyan square. Schematic illustration of the tilt angles of the branches faceted e) with (001) and (101) planes, f) with (001) and (1̅01) planes.

High‐resolution STEM using high‐angle annular dark field (HAADF) imaging of a single nanobristle and its branches along the STO [010] projection direction was performed to reveal the surface morphology of the bristles as shown in **Figure** **3**. Within a single bristle (Figure 3a), two characteristic angles were observed: 73° close to the core and 53° near the tip of the bristle. The shallower branch angle at the bristle tip suggests that the fast growth direction of the anisotropic single crystalline branch changes beyond a critical nanobristle diameter.

To better elucidate the structure of each bristle, a series of atomic‐resolution STEM images were taken from the marked regions in Figure 3a, where the green rectangle (Figure 3b) and the red square (Figure 3c) show branches angled at 53°, and the cyan square (Figure 3d) represents a branch angled at 73° from the plane of the film. Figure 3b−d shows that the branches growing at angles of 53° and 73° are faceted with nearly equal fractions of (001)/(101) and (001)/(1̅01) facets, respectively. The observation that the (001) surface on both of these angled branches are tilted away from the (001) STO surface plane by 18° as shown in Figure 3c,d suggests that the deformation, which consistently causes the 18° tilt of the lattice, is located at the core of the nanobristles.

For anatase TiO_2_, the angle between the (001) and (101) planes is 68.3°. If we consider a branch terminated by equal contributions of (001) and (101) surface as observed and take into account the 18° tilt of the (001) plane with respect to the STO(001) surface plane (Figure 3c,d), one can conclude that the angle between the branch and the STO(001) plane should be 52.1°, as depicted in Figure 3e. A similar analysis for a branch terminated with equal fractions of the (001) and (1_01) surface results in a branch direction of 73.8° (Figure 3f) from the STO(001) plane. These values are in good agreement with the experimentally measured (Figure 3a) angles of 53° and 73°, confirming that the nanobristles of the nanobrush film contain {001} facets with a percentage as high as 50%. This large fraction of (001) terminated surfaces is far larger than that predicted from the equilibrium Wulff shape where >94% of the surface would be expected to be {101} facets.^31^ This observation indicates that these PLD growth conditions result in a structure far from equilibrium. We further note that unlike the conventional approach of the solvothermal synthesis of TiO_2_ nanocrystals,^37^ PLD provides a contamination‐free, controllable, and reproducible process for the direct synthesis of TiO_2_ nanostructures with a high volume of {001} facets. With extensive evidence of fast electron transport in 1D TiO_2_ nanostructures and high reactivity of surface‐engineered TiO_2_ nanoparticles, our vertically aligned single‐crystalline TiO_2_ nanobrushes with a large amount of the high energy {001} facets could have promising applications in many environmental‐ or energy‐related applications, including photocatalysis, photovoltaics, water treatment, and energy storage.

**Figure 4:**
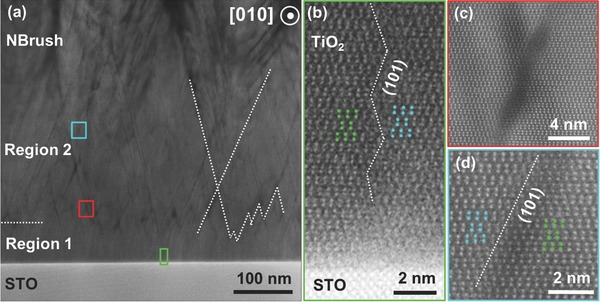
STEM images showing the microstructure evolution of the nanobrush film. a) STEM image of the nanobrush root area; b–d) HAADF images for the regions in (b) the green rectangle, (c) the red square, and (d) the cyan square shown in (a).

To better understand the nature and evolution of the nanobrush architecture, we used STEM imaging to analyze the morphology of different regions, with particular attention to the dark linear features visible mostly in Region 2. **Figure** **4**a shows a low‐magnification STEM image of the transition area between the dense film near the substrate and the porous nanobrush region. A darker zig‐zag boundary dividing the dense part of the film into two sections is clearly visible. Atomic‐resolution STEM images (Figure 4b–d) taken from the regions marked in Figure 4a reveal that the dark lines are domain boundaries oriented along {101} planes (Figure 4d). The crystal lattice of two domains separated by the boundary is shifted by 1/4 unit cell along the <001> direction. Although Region 1 near the STO substrate appears free of these domain boundaries in the low‐magnification image (Figure 4a), the atomic‐scale STEM images (Figure 4b and Figure S1 (Supporting Information)) reveal that these (101) oriented growth defects do form already at the substrate‐film interface and climb up through the first region. Anatase TiO_2_ has an in‐plane lattice constant of 3.784 Å while the lattice constant of STO is 3.905 Å, leading to a lattice mismatch of 3.1%. Thus, the generation of defects could be attributed to strain relaxation of TiO_2_.^43^

The propagation of the domain boundaries along the {101} planes and the progressively higher concentration of these defects with increasing thickness in Region 1 eventually results in a zig‐zag boundary with a maximum defect concentration at a thickness of around 75 nm as one can see in Figure 2c and Figure 4a. Further growth into the Region 2 results in a network of crossing defect planes (see Figure 4a). The dark contrast of these domain boundaries seen in STEM images could originate from the initially formed pores or the disorder in the atomic structure in the boundary region due to the reduced scattering intensity of the atomic columns. Near the apices of the domain boundaries' crossings, the disorder in the crystal structure is significantly larger and the crystal structure was hard to resolve, as shown in Figure 4c.

## 3 Discussion

Combining the growth map in Figure 1 and the STEM observations in Figure 4, it appears that the highly defected areas at the crossing points of the stacking faults serve as effective nucleation sites for the formation of 1D nanostructures at growth conditions with a reduced adatom mobility and suppressed surface diffusion that is locally exacerbated by the defected regions.^44^ Apparently, the defective domain boundaries developed from the (101)‐type stacking faults initially act as surface transport barriers between the root and the surrounding matrix, resulting in dense roots that remain separated from the matrix by the domain boundaries. The root then grows laterally and vertically within mutually inclined (101)‐oriented domain boundaries, leading to the observed conical structure. This is consistent with cross‐sectional SEM images of the nanobrush film (Figure S2, Supporting Information) that shows cleavage of the lower part of the film mostly occurs at the root edges, indicating a relatively weaker bond across the domain boundaries between the root and the film matrix. The density of the roots and the inclined angle of the (101) domain boundaries lead to two adjacent roots eventually meeting at around 280 nm thickness. An anisotropic growth governed by the geometric shadowing effect eventually leads to the formation of columnar structures.

Although it is still not obvious why branches with a high fraction of {001} facets are formed within individual bristles of the nanobrush film, the well‐ordered branch structure itself can be explained within a kinetic aggregation model when incorporating the temperature dependent effects of surface diffusion.^45^, ^46^ At relatively high pressures and/or low substrate temperatures, and thus short surface diffusion lengths, diffusion limited aggregation growth would result in irregular low density dendrite patterns because the arriving species get incorporated in the lattice mostly at exposed surfaces.^7^, ^44^ Instead, at a relatively lower oxygen pressure and intermediate low substrate temperature, planar branch formation can be attributed to a preferred growth of protruding parts generated by ballistic (instead of diffusion limited) aggregation, followed by limited surface diffusion before a next incorporation event terminates the surface diffusion. At the same intermediate substrate temperature, but at even lower pressures, the surface diffusion length is increased due to the less interaction and more kinetic energy, leading to the formation of more dense films. This interpretation is consistent with the observations shown in the growth map of Figure 1, as well as with the evolution of the microstructure extracted from the STEM images that reveal a dense nanobrush root evolving into planar branches (Figure 4). Both data sets suggest that the nanobrush architecture can be preferentially grown when nonequilibrium aggregation and equilibrating surface diffusion processes compete in the presence of a nonuniform distribution of defect sites. A similar growth behavior was recently observed in other oxides, CeO_2_ and Y_2_O_3_, forming vertically aligned nanostructures.^47^ Control of the growth kinetics can thus not only be used to suppress defect formation for growth of atomically precise heterostructures, but also, in the other extreme, to control the formation of novel self‐assembled nanostructured materials. In particular, the growth diagram presented here for a TiO_2_ nanobrush architecture points the way toward nanostructure design by controlling growth kinetics in conjunction with the utilization of growth defects.

## 4 Conclusion

A novel PLD synthesis strategy for 1D nanostructure fabrication of single crystalline anatase TiO_2_ was developed by controlling the surface mobility of the growth species to generate defects and growth instabilities. The nanobrush film features a solid root emanating from crossing points of domain boundaries where a high concentration of defects is located. The surface of the crystalline bristles consists of a high density of the {001} facets, suggesting promising applications where a high chemical activity is desired. The microstructural evolution suggests that the nanobrush formation results from a competition between nonequilibrium aggregation and equilibrating surface diffusion processes. This competition can be altered by controlling the mobility of the growth species through the substrate temperature and oxygen pressure. Thus, this work provides new insight into controllable synthesis of 1D structures, highlighting the role of defect creation in nonequilibrium synthesis. We believe that the comprehensive understanding of the growth mechanism of anatase TiO_2_ nanobrush formation makes this growth strategy attractive for a broad range of materials and architectures.

## 5 Experimental Section

Anatase‐phase TiO_2_ films were grown on TiO_2_‐terminated (001) STO substrates by PLD. The STO substrates were chemically etched by buffered HF for 30 s and then thermally treated at 1100 °C in air for 1.5 h. TiO_2_ nanobrush films with a thickness of 1.5 µm were deposited with a KrF excimer laser using a TiO_2_ polycrystalline target. In order to check the viability of growing single crystalline nanoarchitectures by PLD, various growth conditions were explored. In order to control the surface diffusion of adatoms, the substrate temperature (*T* = 400–600 °C) and oxygen background pressure (*p*(O_2_) = 50–200 mTorr) were systematically varied. We grew TiO_2_ films with a fixed number of laser pulses (50 000 pulses), and the typical deposition rate was 0.3 Å per laser pulse. For structural characterization, XRD was carried out using a high‐resolution X‐ray diffractometer with Cu *K*_α1_ radiation. The plan view morphology of samples was characterized by SEM. High‐angle annular dark‐field (HAADF) imaging was carried out in Na ion UltraSTEM200 operated at 200 kV. The microscope is equipped with a cold field‐emission gun and an aberration corrector for sub‐angstrom resolution. Inner/outer angles of 70/240 mrad were used for HAADF imaging. The convergence semi‐angle for electron probe was set to 30 mrad.

## Supporting information

Supplementary
