# Supplementary material for: Kinetically Controlled Fabrication of Single‐Crystalline TiO2 Nanobrush Architectures with High Energy {001} Facets
Source: Adv Sci (Weinh). 2017 Apr 5;4(8):1700045. doi: 10.1002/advs.201700045 (PMC5566339; doi:10.1002/advs.201700045)
Supplement: Supplementary file 1 — Supplementary [file ADVS-4-na-s001.pdf]

Copyright WILEY-VCH Verlag GmbH & Co. KGaA, 69469 Weinheim, Germany, 2017.

ADVANCED SCIENCE
Open Access

Supporting Information

for *Adv. Sci.,* DOI: 10.1002/advs.201700045

Kinetically Controlled Fabrication of Single-Crystalline TiO_2 Nanobrush Architectures with High Energy {001} Facets

*Lisha Fan, Xiang Gao, Dongkyu Lee, Er-Jia Guo, Shinbuhm Lee, Paul C. Snijders, Thomas Z. Ward, Gyula Eres, Matthew F. Chisholm, and Ho Nyung Lee**

Copyright WILEY-VCH Verlag GmbH & Co. KGaA, 69469 Weinheim, Germany, 2016.

Supporting Information

Kinetically Controlled Fabrication of Single-Crystalline TiO_2 Nanobrush Architectures with High Energy {001} Facets

*Lisha Fan, Xiang Gao, Dongkyu Lee, Er Jia Guo, Shinbuhm Lee, Paul C. Snijders, Thomas Z. Ward, Gyula Eres, Matthew F. Chisholm, Ho Nyung Lee**

1. STEM characterization of the film-substrate interface shows uniform distributed defects of stacking faults generated at the film-substrate interface due to lattice mismatch between the TiO_2 anatase film and the STO substrate.

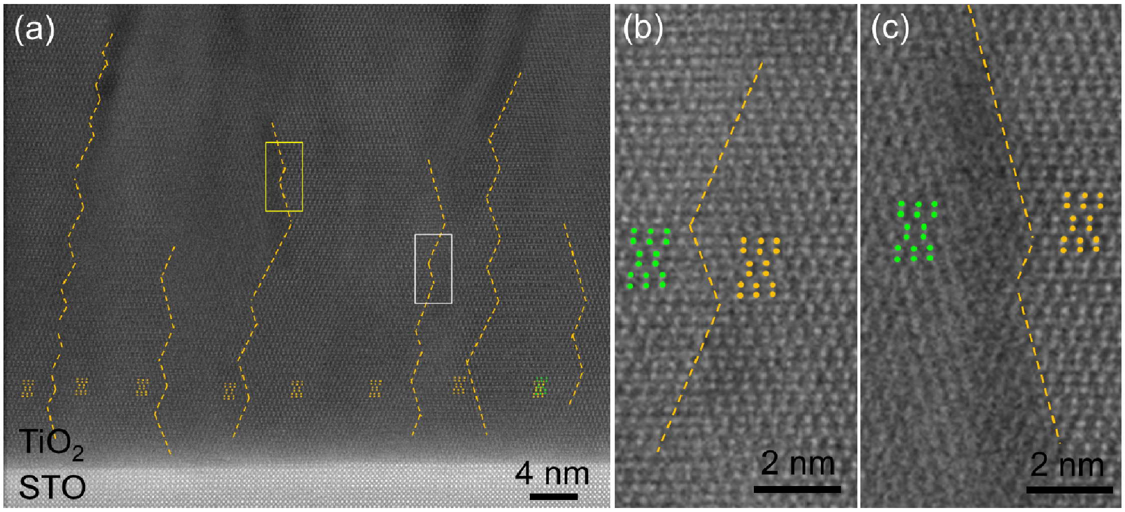

Figure S1. (a) A STEM image of the film-substrate interface region of a TiO_2 nanobrush sample. High magnification STEM images for the regions (b) in the white rectangle and (c) the yellow rectangle.

2. SEM characterization of the nanobrush root region shows the cleavage of the film occurs at the root-matrix boundary, suggesting the roots are loosely bounded with the surrounding matrix.

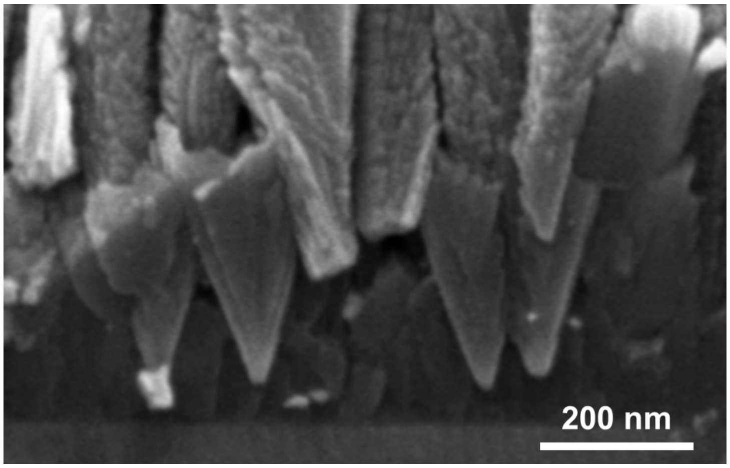

Figure S2. A SEM image of the conical roots of TiO_2 nanobrushes.
